# Weekly somapacitan had no adverse effects on glucose metabolism in adults with growth hormone deficiency

**DOI:** 10.1007/s11102-022-01283-3

**Published:** 2022-11-15

**Authors:** Yutaka Takahashi, Beverly M. K. Biller, Hidenori Fukuoka, Ken K. Y. Ho, Michael Højby Rasmussen, Navid Nedjatian, Claus Sværke, Kevin C. J. Yuen, Gudmundur Johannsson

**Affiliations:** 1 Department of Diabetes and Endocrinology, Nara Medical University, 840 Shijo-Cho, Kashihara, Nara 634-8522 Japan; 2 Neuroendocrine & Pituitary Tumor Clinical Center, Massachusetts General Hospital and Harvard Medical School, Boston, MA USA; 3 Division of Diabetes and Endocrinology, Kobe University Hospital, Kobe, Japan; 4 Garvan Institute of Medical Research, St. Vincent’s Hospital, Sydney, Australia; 5 UNSW Sydney, Sydney, Australia; 6 Global Development, Novo Nordisk A/S, Søborg, Denmark; 7 Global Medical Affairs–Rare Endocrine Disorders, Novo Nordisk Health Care AG, Zurich, Switzerland; 8 Barrow Pituitary Center, Barrow Neurological Institute and St. Joseph’s Hospital and Medical Center, University of Arizona College of Medicine, Phoenix, AZ USA; 9 Creighton School of Medicine, Phoenix, AZ USA; 10 Institute of Medicine, Sahlgrenska Academy, University of Göteborg, Göteborg, Sweden; 11 Department of Endocrinology, Sahlgrenska University Hospital, Göteborg, Sweden

**Keywords:** Adult growth hormone deficiency, Somapacitan, Glucose metabolism, Growth hormone, Insulin sensitivity, Growth hormone replacement therapy

## Abstract

**Purpose:**

The long-term effects of long-acting growth hormone (LAGH) analogues on glucose metabolism in adult growth hormone deficiency (AGHD) are not known. We investigated the impact of LAGH somapacitan, administered once-weekly, on glucose metabolism in patients with AGHD.

**Methods:**

In post hoc-defined analyses, we compared the effects of somapacitan with daily growth hormone (GH) and placebo on fasting plasma glucose (FPG), glycated hemoglobin (HbA1c), fasting insulin, homeostasis model assessment of insulin resistance (HOMA-IR) and beta-cell function (HOMA-β) in patients with AGHD across a unique data set from three phase 3 randomized controlled trials (REAL 1, REAL 2 and REAL Japan).

**Results:**

No new cases of diabetes mellitus were reported with somapacitan. Among GH-naïve patients (n = 120 somapacitan, n = 119 daily GH), higher changes from baseline in FPG, HOMA-IR and fasting insulin levels were observed with daily GH versus somapacitan at 34 weeks, but not at 86 weeks. HbA1c and HOMA-β did not differ between groups at either timepoint. Among treatment-naïve patients, sex, age, fasting insulin, glucose tolerance status and body mass index did not influence changes in glucose metabolism. In previously treated patients (REAL 1 extension: n = 51 somapacitan, n = 52 daily GH; REAL 2: n = 61 and n = 31, respectively; REAL Japan: n = 46 and n = 16, respectively), the difference in changes from baseline were not statistically significant between somapacitan and daily GH for any glucose metabolism parameters.

**Conclusions:**

Somapacitan, compared with daily GH, did not adversely affect glucose metabolism up to 86 weeks in a large cohort of treatment-naïve or previously treated patients with AGHD.

Trial registrations (date of registration): NCT02229851 (2 September 2014), NCT02382939 (3 March 2015), NCT03075644 (7 March 2017).

**Supplementary Information:**

The online version contains supplementary material available at 10.1007/s11102-022-01283-3.

## Introduction

Untreated adult growth hormone deficiency (AGHD) is associated with increased body fat, an adverse lipid profile, increased cardiovascular disease risk, impaired glucose tolerance and metabolic syndrome [1, 2]. Patients with AGHD also have a compromised quality of life and a decreased capacity for exercise [3]. Growth hormone replacement therapy (GHRT) in AGHD improves body composition (reduced fat mass, increased lean mass and muscle strength), bone mineral density and cardiovascular risk markers (increased high-density lipoprotein cholesterol and reductions in low-density lipoprotein cholesterol, C-reactive protein, diastolic blood pressure and carotid intima-media thickness), as well as improving quality of life [4, 5].

Given that growth hormone (GH) regulates glucose homeostasis, untreated patients with AGHD are predisposed to an increased risk of altered glucose metabolism, characterized by insulin resistance and fasting hyperinsulinemia [6–8]. Most studies have shown that there is a slight increase in glucose levels and insulin resistance with GHRT [8], especially during the early phase of GH substitution [9] and when higher GH doses are used [10]. A recent systematic review that included both open-label and randomized controlled studies suggested that negative effects of GHRT on glucose homeostasis parameters were seen with shorter (defined as 6–12 months), but not longer, durations of GHRT, although fasting plasma glucose (FPG) remained elevated in some studies [11]. However, long-term observational studies have suggested that the incidence of diabetes mellitus in patients with AGHD receiving GHRT does not appear to increase [12–14] or that any increased incidence is observed in patients with underlying risk factors for diabetes mellitus [6, 15]. Known risk factors for diabetes mellitus include older age, greater body mass index (BMI) and higher degrees of insulin resistance [16, 17].

GH increases glucose production through gluconeogenesis and glycogenolysis in the liver and kidney [18]. GH also induces insulin resistance through both direct effects on the insulin receptor and stimulation of lipolysis, resulting in the release of free fatty acids from adipose tissue to the circulation [8, 18]. Continuous high exposure to GH, such as that seen in acromegaly, is also associated with hyperinsulinemia, impaired glucose tolerance and diabetes mellitus [8]. Therefore, despite the lack of clear evidence, there are concerns about increased insulin resistance and impaired glucose metabolism in patients receiving GHRT, particularly in those with underlying risk factors for glucose intolerance. As such, current treatment guidelines recommend monitoring glucose metabolism parameters (FPG and glycated hemoglobin [HbA1c]) in adults receiving GHRT [19].

Somapacitan (Sogroya®; Novo Nordisk, Denmark) is a once-weekly, long-acting GH (LAGH) derivative approved for the treatment of AGHD. In three randomized controlled trials in adults with GHD (REAL 1, REAL 2 and REAL Japan), somapacitan was shown to have similar efficacy and safety to daily GH [20–22]. During these three trials, no new cases of diabetes mellitus were reported in patients treated with somapacitan.

The objective of the current study was to further investigate the long-term effects of somapacitan on glucose metabolism and insulin sensitivity in patients with AGHD. This paper reports the results from all three randomized controlled studies, giving a unique, large data set to address the effects of LAGH somapacitan on glucose metabolism over treatment periods from 26 to 86 weeks.

## Methods

### Trials used for the analyses: study designs and comparator groups

REAL 1 (NCT02229851), REAL 2 (NCT02382939) and REAL Japan (NCT03075644) were multicenter, prospective, randomized, parallel-group phase 3 studies that compared somapacitan with daily GH (Norditropin®; Novo Nordisk, Denmark). REAL 1 investigated the efficacy and safety of somapacitan; REAL 2 was a safety and tolerability study; and REAL Japan was primarily a safety study, with secondary efficacy endpoints. Individual study designs are shown in Supplementary Figure S1. Detailed methods for each of these trials have previously been reported [20–22]. Each study was approved by the relevant local and national ethics committee and conducted with written consent from all patients in accordance with the Declaration of Helsinki and the International Conference on Harmonization Guidelines for Good Clinical Practice.

Patients with a diagnosis of adult- or childhood-onset AGHD were eligible for the trials. The permitted age range was 23–79 years in REAL 1 and 18–79 years in REAL 2 and REAL Japan. Patients in REAL 1 were GH-naïve, defined as no prior GH treatment (true naïve) or no GH treatment for ≥ 180 days before study start, whereas patients in REAL 2 and REAL Japan had received treatment with GH for ≥ 6 months prior to screening [20–22]. Patients with diabetes mellitus could be included in REAL 1 and REAL 2 only if they met the following criteria: diabetes mellitus diagnosed clinically ≥ 6 months prior to screening; on stable oral antidiabetic treatment, unchanged for ≥ 90 days prior to screening; no history of use of injectable anti-diabetic agents; HbA1c < 7.0% at screening according to the central laboratory; no diabetes-related comorbidities (as judged by the investigator) at screening; and no evidence of proliferative retinopathy or severe nonproliferative diabetic retinopathy ≤ 90 days prior to randomization [20, 21]. By contrast, patients with diabetes mellitus were excluded from REAL Japan [22].

In the REAL 1 and REAL 2 trials, both somapacitan and daily GH were dose-titrated for 8 weeks to achieve an insulin-like growth factor I (IGF-I) standard deviation score (SDS) within the normal range, and then administered at a fixed dose (which could be reduced if necessary for safety reasons). The titration period for REAL Japan was 20 weeks.

In the main part of the REAL 1 study, 300 GH-naïve patients were randomized and exposed to once-weekly somapacitan (n = 120), once-weekly placebo (n = 61) or daily GH (n = 119) for 34 weeks. After the dose-titration period, the mean (standard deviation [SD]) dose was 2.56 (1.48) mg/week for somapacitan and 0.33 (0.19) mg/day for daily GH.

During the 52-week REAL 1 extension, 114 patients who had received somapacitan in the main phase continued on somapacitan (somapacitan/somapacitan group). All patients who had received placebo and continued in the extension were switched to somapacitan (placebo/somapacitan group, n = 55). Patients who had received daily GH were re-randomized either to receive somapacitan (daily GH/somapacitan group, n = 51) or continue daily GH (daily GH/daily GH group, n = 52) (Supplementary Figure S1). After a 1-week washout period, dose titration was again performed for 8 weeks. After titration, mean doses (SD) were 2.35 (1.30) mg/week for the somapacitan/somapacitan group, 0.28 (0.16) mg/day for the daily GH/daily GH group and 2.66 (1.37) mg/week for the daily GH/somapacitan group.

For the REAL 1 extension phase, comparisons of change from baseline (week 0) for the ‘GH-naïve AGHD patients’ were between the somapacitan/somapacitan group and daily GH/daily GH group. Comparisons for the ‘previously treated AGHD patients’ (i.e., for the change between week 34 and week 86) were between the daily GH/somapacitan group and daily GH/daily GH group (see Table 1 for an explanation of comparator groups).

**Table 1:** Patient groups used for between-treatment comparisons

Patient groups used for comparisons of treatment-naïve patients		
REAL 1 (34 weeks)		
Somapacitan, n = 120	Daily GH, n = 119	Placebo, n = 61
REAL 1 main + extension (86 weeks)		
Somapacitan/somapacitan, n = 120	Daily GH/daily GH, n = 52	–
Patient groups used for comparisons of previously treated patients		
REAL 2 (26 weeks)		
Somapacitan, n = 61	Daily GH, n = 31	
REAL Japan (52 weeks)		
Somapacitan, n = 46	Daily GH, n = 16	
REAL 1 extension (52 weeks)		
Daily GH/somapacitan, n = 51	Daily GH/daily GH, n = 52	

*GH* growth hormone

In REAL 2, 92 patients were randomized to receive once-weekly somapacitan (n = 61) or daily GH (n = 31) for 26 weeks (Supplementary Figure S1). After the dose-titration period, the mean dose (SD) was 1.96 (1.45) mg/week for somapacitan and 0.20 (0.14) mg/day for daily GH.

In REAL Japan, 62 patients were randomized to receive once-weekly somapacitan (n = 46) or daily GH (n = 16) for 52 weeks. Mean (SD) prescribed doses after titration were 1.78 (1.06) mg/week for somapacitan and 0.12 (0.08) mg/day for daily GH. Patients from REAL 2 and REAL Japan were categorized as ‘previously treated AGHD patients’ for the purposes of analyses (Table 1).

### Assessment of glucose metabolism

The effects of somapacitan and daily GH on glucose metabolism in each trial were assessed by measuring FPG and HbA1c. Insulin resistance was assessed by determining glucose to insulin relationships (which included measuring fasting insulin) to derive homeostasis model assessment insulin resistance (HOMA-IR) and steady-state beta-cell function (HOMA-β). HOMA-IR and HOMA-β were calculated as follows:

$$ \text{HOMA-IR} (\%)= \frac{\mathrm{Fasting\, insulin }\,\left(\mathrm{pmol}/\mathrm{L}\right)\,\mathrm{ x }\,\frac{1}{6}\,\mathrm{ x \,FPG }\,(\mathrm{mmol}/\mathrm{L})}{22.5}$$

$$\text{HOMA-}\upbeta (\mathrm{\%}) =\frac{\left[20\, \mathrm{ x\, fasting\, insulin }\,\left(\mathrm{pmol}/\mathrm{L}\right)\,\mathrm{ x }\,\frac{1}{6} \right]}{[\mathrm{FPG }\,\left(\mathrm{mmol}/\mathrm{L}\right) - 3.5]}$$

If FPG was ≤3.5mmol/L then the response was set to missing.

### Data collection and analysis

The timing of blood sampling for glucose metabolism differed across the three studies (Supplementary Figure S1). However, all blood samples used in the current study for comparison with daily GH (REAL 1 main and its extension at weeks 34 and 87, respectively, REAL 2 at week 26 and REAL Japan at week 52) were taken 4 days after somapacitan dosing, the point at which IGF-I levels have been shown to correspond with mean IGF-I levels over the week [23]. Details on the collection and analysis of samples are provided in the publication reporting each trial [20–22]. In each trial, analyses were performed by a central laboratory.

### Baseline characteristics

In these analyses, data from both GH-naïve and previously treated AGHD patients were used. Baseline characteristics of the comparator groups, including the number of patients with diabetes mellitus, are presented in Table 2. Within each trial, baseline characteristics (including glucose parameters) were generally similar between the treatment groups.

**Table 2:** Baseline characteristics of patients included in this analysis

	Treatment naïve			Previously treated					
	REAL 1 (34 weeks)			REAL 1 extension (week 34 to week 86)		REAL 2 (26 weeks)		REAL JAPAN (52 weeks)	
	Somapacitan (n = 120)	Daily GH (n = 119)	Placebo (n = 61)	Daily GH/ somapacitan^a^ (n = 51)	Daily GH/ daily GH^a^ (n = 52)	Somapacitan (n = 61)	Daily GH (n = 31)	Somapacitan (n = 46)	Daily GH (n = 16)
Age, years, mean (SD)	44.6 (14.3)	45.7 (15.3)	45.0 (15.7)	44.0 (15.9)	47.0 (14.6)	48.1 (16.2)	51.7 (17.1)	54.1 (12.1)	49.3 (11.5)
Female, n (%)	62 (51.7)	61 (51.3)	32 (52.5)	27 (52.9)	28 (53.8)	28 (45.9)	14 (45.2)	22 (47.8)	7 (43.8)
GHD onset, n (%)									
Childhood – idiopathic	21 (17.5)	21 (17.6)	13 (21.3)	11 (21.6)	9 (17.3)	6 (9.8)	3 (9.7)	4 (8.7)	1 (6.3)
Childhood – organic	17 (14.2)	12 (10.1)	7 (11.5)	3 (5.9)	6 (11.5)	18 (29.5)	7 (22.6)	5 (10.9)	1 (6.3)
Adulthood	82 (68.3)	86 (72.3)	41 (67.2)	37 (72.5)	37 (71.2)	37 (60.7)	21 (67.7)	37 (80.4)	14 (87.5)
Region, n (%)									
Japan	18 (15.0)	18 (15.1)	10 (16.4)	8 (15.7)	9 (17.3)	11 (18.0)	6 (19.4)	46 (100.0)	16 (100.0)
Rest of world	102 (85.0)	101 (84.9)	51 (83.6)	43 (84.3)	43 (82.7)	50 (82.0)	25 (80.6)	0	0
Mean BMI, kg/m^2^ (SD)	27.9 (6.3)	27.7 (6.2)	26.1 (6.4)	27.2 (6.2)	28.2 (6.3)	28.6 (5.0)	28.5 (5.6)	26.4 (6.7)	24.8 (3.7)
Mean waist circumference, cm (SD)	93.9 (16.6)	94.3 (15.1)	88.2 (14.5)	92.9 (16.5)	96.2 (16.2)	NA	NA	NA	NA
Diabetes mellitus, n (%)	7 (5.8)	6 (5.0)	3 (4.9)	2 (3.9)	3 (5.8)	0	1 (3.2)	0	0
IGF-I SDS, mean (SD)	− 2.58 (1.21)	− 2.53 (1.18)	− 2.68 (1.29)	− 2.72 (1.10)	− 2.43 (1.25)	0.28 (1.50)	0.91 (1.24)	0.64 (0.72)	0.88 (0.82)
FPG, mmol/L, mean (SD)	4.94 (0.67)	5.03 (0.70)	4.91 (0.59)	5.03 (0.61)	5.29 (1.11)	5.34 (0.69)	5.39 (0.72)	5.22 (0.55)	5.30 (0.76)
Fasting serum insulin, pmol/L, mean (SD)	79.9 (76.59)	73.6 (52.0)	62.1 (43.09)	101.6 (76.23)	111.2 (102.93)	90.2 (79.18)	77.1 (54.75)	78.7 (64.6)	90.2 (64.8)
HbA1c, %, mean (SD)	5.43 (0.43)	5.43 (0.43)	5.48 (0.34)	5.49 (0.39)	5.50 (0.53)	5.42 (0.42)	5.48 (0.41)	5.73 (0.38)	5.71 (0.49)
HOMA-β, mean (SD)	198.53 (198.63)	188.61 (255.48)	165.97 (147.49)	228.23 (153.45)	241.47 (216.57)	162.18 (116.93)	139.13 (89.83)	131.9 (102.91)	142.79 (82.05)
HOMA IR, % mean (SD)	3.07 (3.05)	2.86 (2.29)	2.33 (1.72)	3.94 (3.23)	4.56 (4.77)	3.76 (3.54)	3.24 (2.76)	2.69 (2.31)	3.24 (2.65)

^**a**^These two groups are subgroups of the daily GH group in REAL 1. Patients receiving daily GH in the main phase were re-randomized at week 34 to receive either daily GH or somapacitan in the extension phase. Baseline for these patients is week 34 (end of the main phase/start of the extension phase)

*BMI* body mass index, *FPG* fasting plasma glucose, *GH* growth hormone, *GHD* growth hormone deficiency, *HbA1c* glycated haemoglobin, *HOMA-β* steady state beta-cell function, *HOMA-IR* homeostasis model assessment insulin resistance, *IGF-I* insulin-like growth factor I, *NA* not available, *SD* standard deviation, *SDS* standard deviation score

### Statistical analysis

Post hoc-defined statistical analyses examined the difference in absolute or relative changes from baseline (mean, SD; *P*-value) between treatment groups on FPG, HbA1c, fasting insulin, HOMA-IR and HOMA-β. The results were reported as estimated treatment differences (somapacitan – daily GH, for FPG and HbA1c) or estimated treatment ratios (ratio of somapacitan to daily GH, for fasting serum insulin, HOMA-IR and HOMA-β).

Changes from baseline to subsequent week measurements were analyzed using a mixed model for repeated measurements (MMRM), with treatment, type of GHD onset (adult or child), sex and region (Japan versus all other countries) as factors, and baseline value as a covariate, all nested within week as a factor. For REAL 1 (main and main plus extension) and REAL 2, diabetes mellitus status and sex by region by diabetes mellitus interaction were also included as factors.

Data on fasting serum insulin, HOMA-IR and HOMA-β were log-transformed for all values at all visits before analysis. From the MMRM, the treatment differences at a specific week between somapacitan and daily GH were estimated, and the corresponding 95% confidence intervals and *P*-values were calculated for each endpoint. No adjustment for multiplicity was performed.

To examine whether the effects of somapacitan and daily GH on glucose metabolism differed according to patients’ baseline characteristics and risk factors, further subgroup analyses were explored in treatment-naive patients from the REAL 1 main phase, based on sex, age (< 40, 40–59 and ≥ 60 years), baseline fasting serum insulin (normal [≥ 14– ≤ 208 pmol/L] or abnormal), baseline glucose tolerance (normal: FPG < 5.6 mmol/L and HbA1c < 5.7%, or prediabetes: FPG ≥ 5.6 mmol/L or HbA1c ≥ 5.7%) [16, 24] and baseline BMI (< 30 kg/m^2^, ≥ 30 kg/m^2^) [25].

## Results

In GH-naïve patients in REAL 1, mean baseline IGF-I SDS was below − 2.5 in all treatment groups (Table 2). Mean IGF-I SDS increased after 34 weeks of treatment with somapacitan and daily GH to similar values [20]. In previously treated patients, mean baseline IGF-I SDS values were maintained throughout each trial, were similar and within the normal range in the somapacitan and daily GH groups at the end of each trial (Supplementary Table S1) [20–22]**.** These findings indicate that GH exposure was similar between treatment arms in each trial.

No new cases of diabetes mellitus were reported during the studies in the patients treated with somapacitan. Among patients treated with daily GH, two patients in REAL 1 and one patient in REAL Japan were diagnosed with diabetes mellitus during the trial.

### Treatment-naïve patients (REAL 1 main phase and REAL 1 main plus extension)

The REAL 1 study design provided the opportunity for comparing treatment effects between treatment groups during both the main phase and extension phase. During the main phase (the only one that included placebo), there were no statistically significant differences in change from baseline to week 34 between the somapacitan and placebo treatment arms for any of the glucose parameters.

**Fig. 1:**
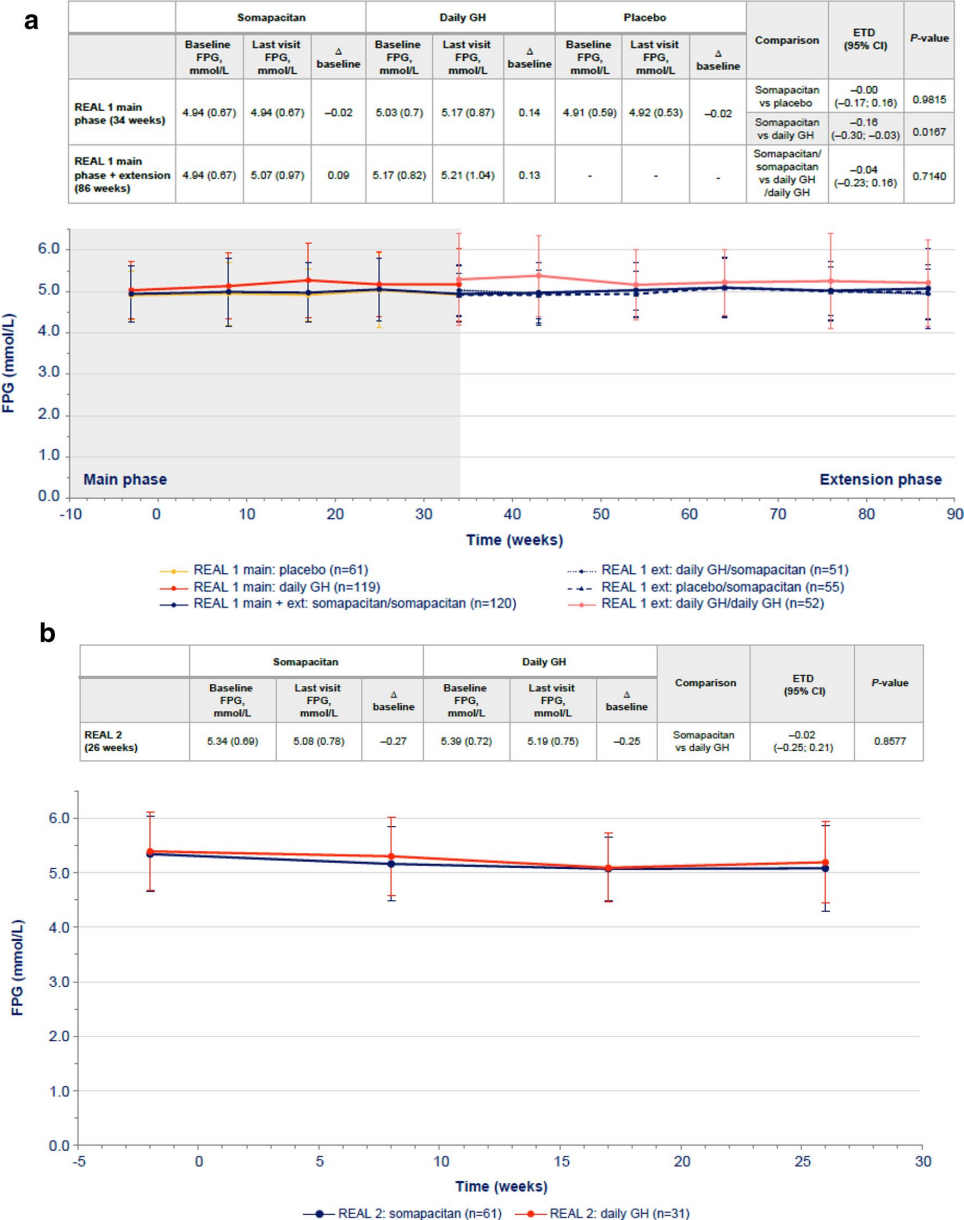

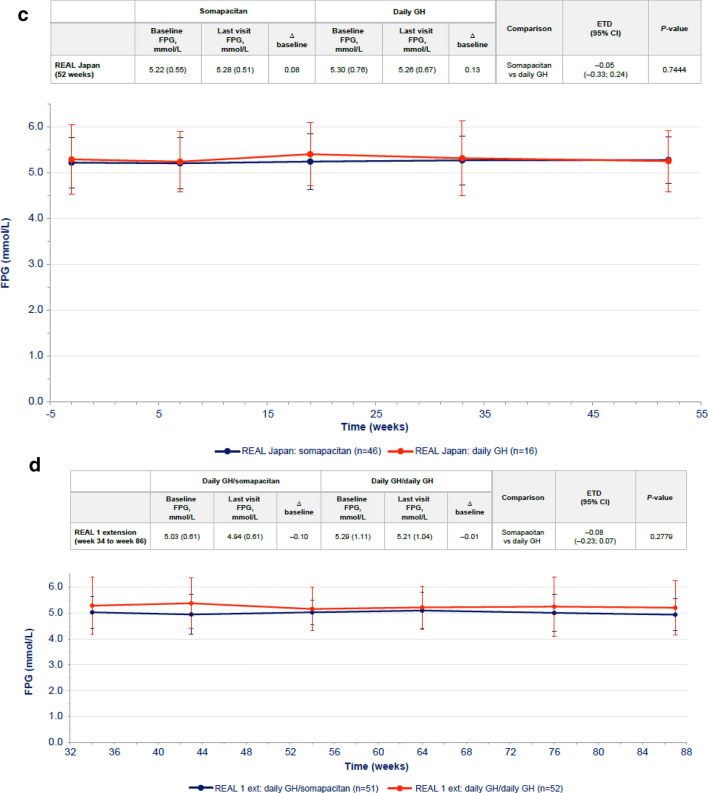
FPG over time in **a** treatment-naïve patients from REAL 1 study (main phase and main + extension phases) and in previously treated patients from **b** REAL 2, **c** REAL Japan and **d** REAL 1 extension phase following re-randomization. Data within tables and figures are mean (SD) (represented by points and error bars). Baseline and last visit values are observed values. Relative changes are shown as differences. Relative changes and ETDs were obtained using a mixed effects model. For patients in the REAL 1 study (**a**), there was a 1-week washout period between the main and extension phases; patients receiving daily GH in the main phase (red) were re-randomized at week 34 to receive either daily GH (pink) or somapacitan (dotted blue) in the extension phase. Δ baseline, change from baseline; *CI* confidence interval, *ETD* estimated treatment difference, *Ext* extension, *FPG* fasting plasma glucose, *GH* growth hormone, *SD* standard deviation

**Fig. 2:**
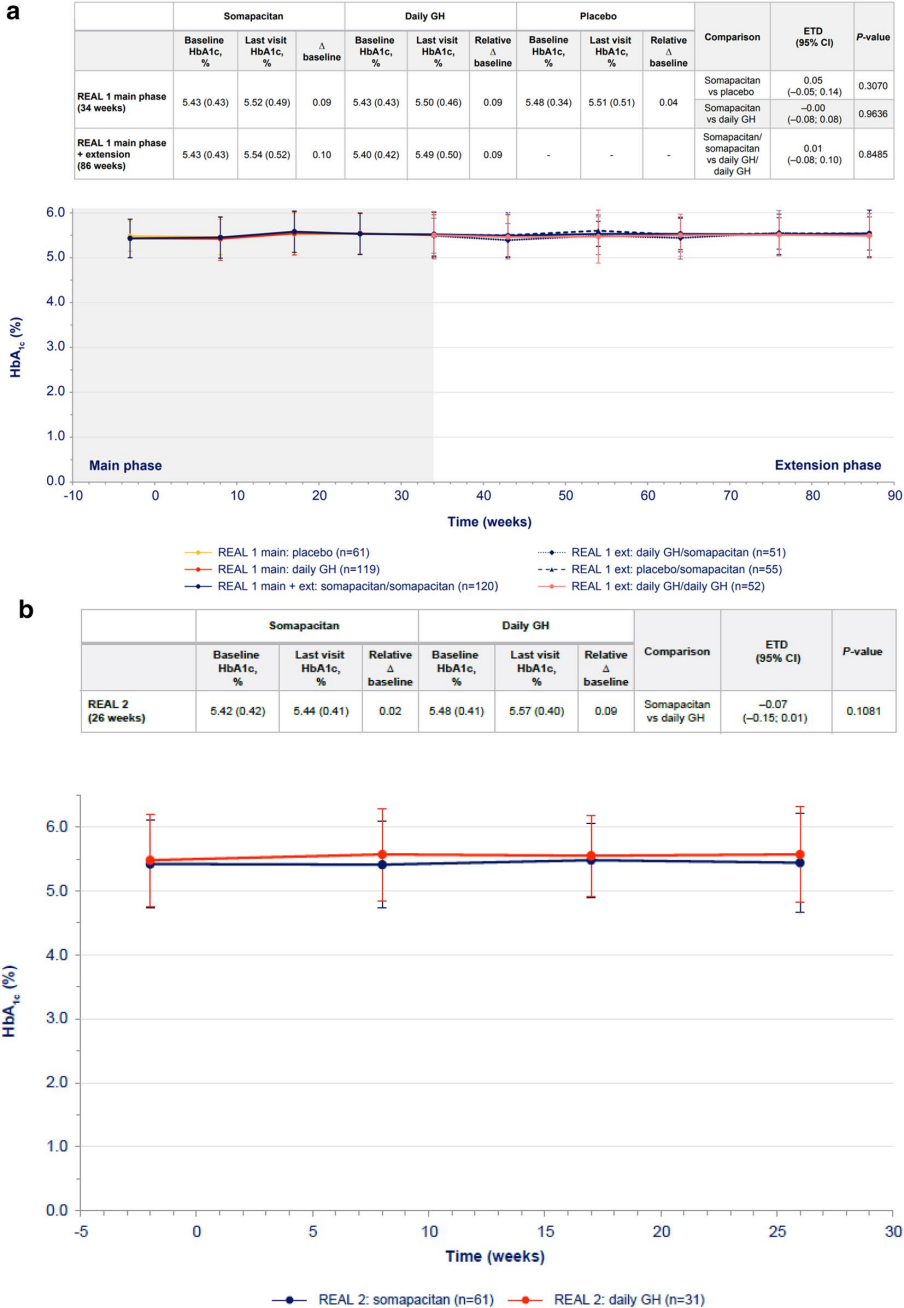

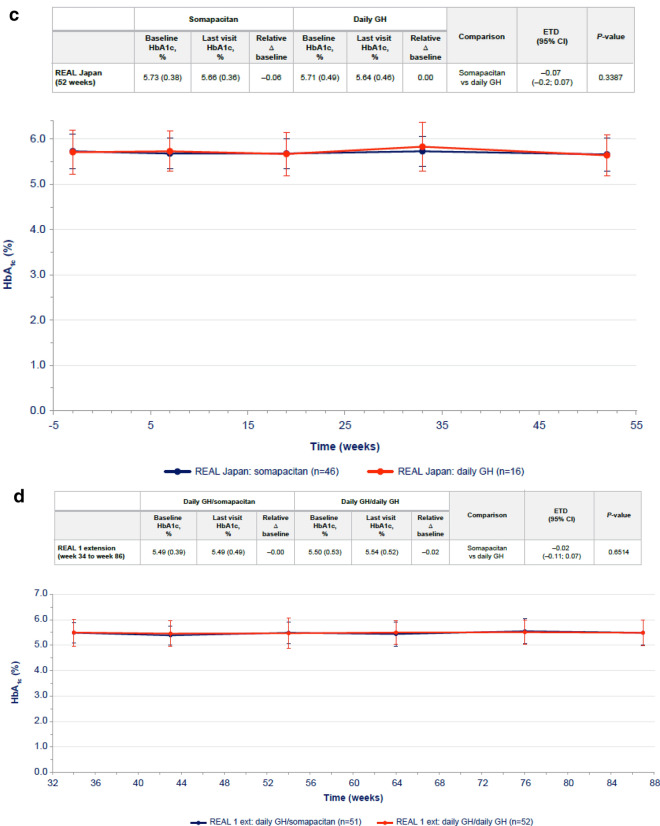
HbA1c values vs time (mean ± SD) in **a** treatment-naïve patients from REAL 1 study (main phase and main + extension phase) and in previously treated patients from **b** REAL 2, **c** REAL Japan and **d** REAL 1 extension phase study following re-randomization of treatment. Data within tables and figures are mean (SD) (represented by points and error bars). Baseline and last visit values are observed values. Relative changes are shown as differences. Relative changes and ETDs were obtained using a mixed effects model. For patients in the REAL 1 study (**a**), there was a 1-week washout period between the main and extension phases; patients receiving daily GH in the main phase (red) were re-randomized at week 34 to receive either daily GH (pink) or somapacitan (dotted blue) in the extension phase. Δ baseline, change from baseline; *CI* confidence interval, *ETD* estimated treatment difference, *Ext* extension, *GH* growth hormone, *HbA1c* glycated hemoglobin, *SD* standard deviation

In GH-naïve patients, changes from baseline in FPG levels were transient. Changes were higher with daily GH than with somapacitan at week 34, but after 86 weeks of treatment, the difference was not statistically significant (Fig. 1a). There were no statistically significant differences in change in HbA1c between the somapacitan and daily GH groups in either phase of the trial (Fig. 2a).

**Fig. 3:**
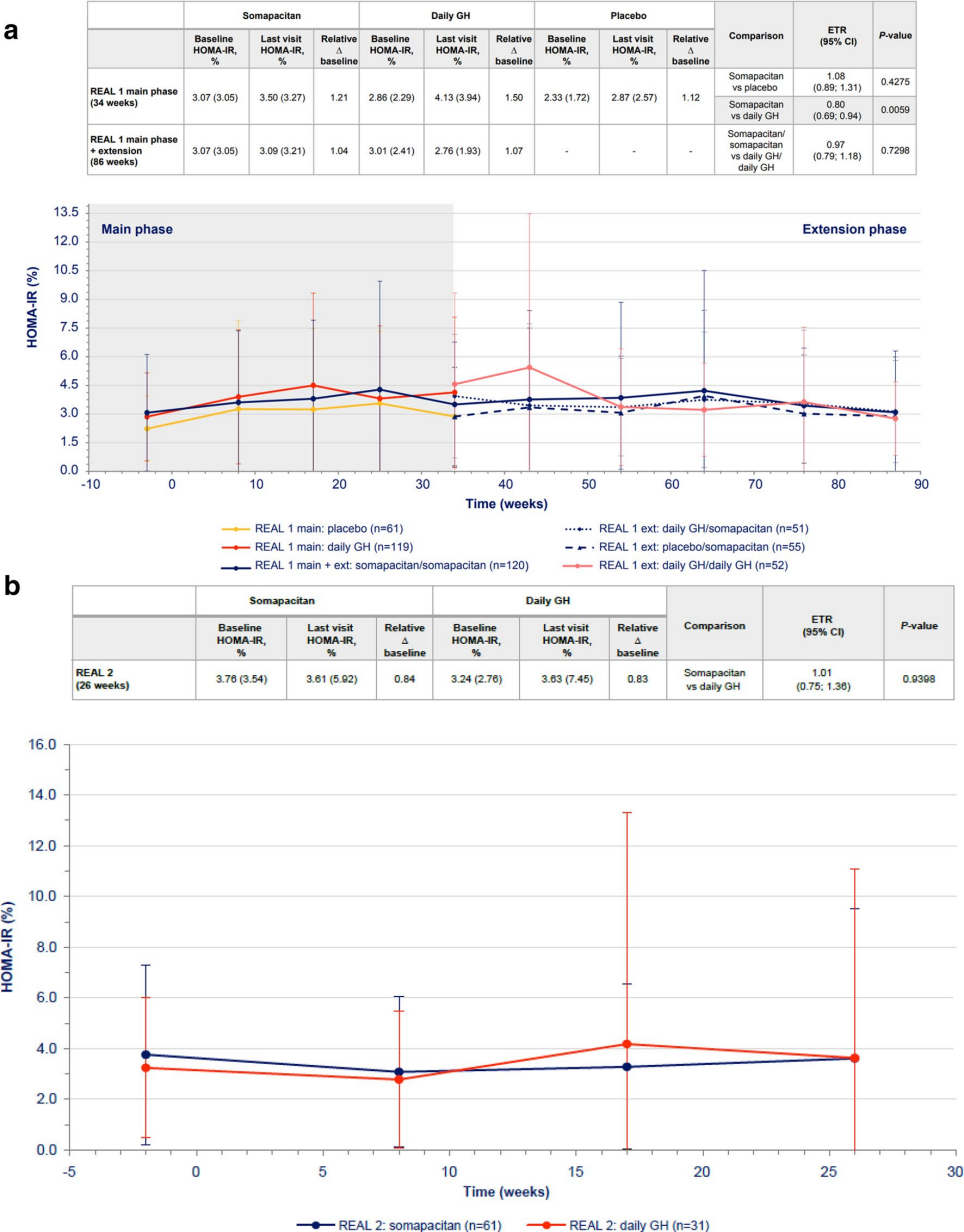

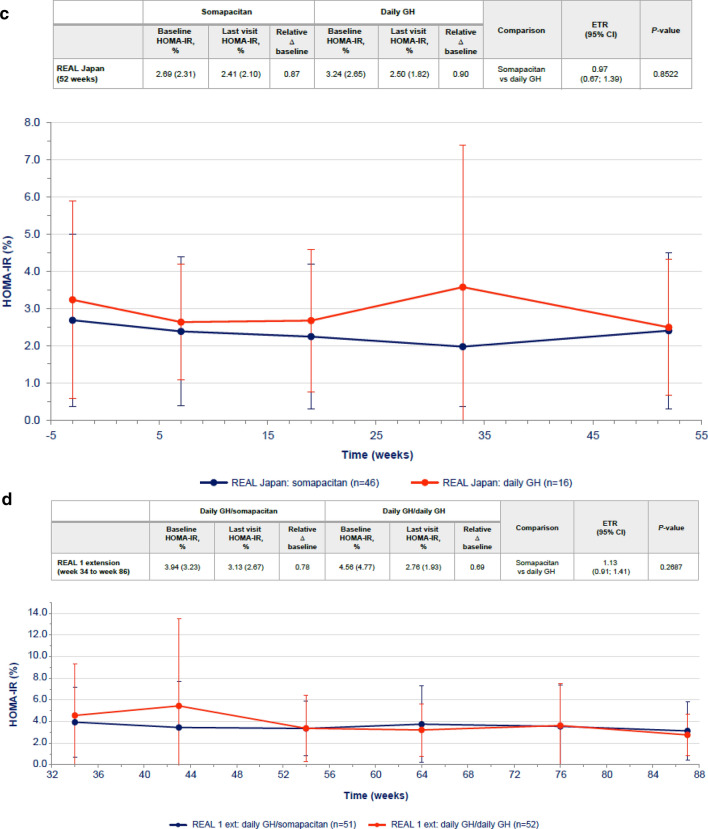
HOMA-IR values over time (mean ± SD) in **a** treatment-naïve patients from REAL 1 study (main phase and main + extension phases) and in previously treated patients from **b** REAL 2, **c** REAL Japan and **d** REAL 1 extension phase study following re-randomization of treatment. Data within tables and figures are mean (SD) (represented by points and error bars). Baseline and last visit values are observed values. Relative changes are shown as ratios. Relative changes and ETRs were obtained using a mixed effects model. For patients in the REAL 1 study (**a**), There was a 1-week washout period between the main and extension phases; patients receiving daily GH in the main phase (red) were re-randomized at week 34 to receive either daily GH (pink) or somapacitan (dotted blue) in the extension phase. Δ baseline, change from baseline; *CI* confidence interval, *ETR* estimated treatment ratio, *Ext* extension, *GH* growth hormone, *HOMA-IR* homeostasis model assessment insulin resistance, *SD* standard deviation

Transient differences in HOMA-IR were observed between somapacitan and daily GH at week 34, with higher values reported for daily GH (Fig. 3a), but the difference was not statistically significant at 86 weeks. Similarly, transient differences in fasting serum insulin were observed between somapacitan and daily GH at 34 weeks, but the difference was not statistically significant at 86 weeks (Table 3). No statistically significant differences in HOMA-β were observed between the somapacitan and daily GH groups in either phase of the trial (Supplementary Table S2).

**Table 3:** Changes from baseline in fasting serum insulin and comparisons (estimated treatment ratio) between treatment groups

Treatment-naïve patients												
	Somapacitan			Daily GH			Placebo			Comparison	ETR (95% CI)	*P*-value
	Baseline FSI, pmol/L	Last visit FSI, pmol/L	Relative Δ baseline	Baseline FSI, pmol/L	Last visit FSI, pmol/L	Relative Δ baseline	Baseline FSI, pmol/L	Last visit FSI, pmol/L	Relative Δ baseline			
REAL 1 main phase (34 weeks)	79.9 (76.59)	92.1 (80.36)	1.23	73.6 (52.0)	103.5 (87.57)	1.45	62.1 (43.09)	75.6 (68.85)	1.10	Somapacitan vs placebo	1.12 (0.94; 1.35)	0.2079
										Somapacitan vs daily GH	0.85 (0.73; 0.99)	0.0313
REAL 1 main phase + extension (86 weeks)	79.9 (76.59)^a^	78.6 (65.67)	1.05	74.8 (52.57)	69.6 (45.08)	1.04	–	–	–	Somapacitan/somapacitan vs daily GH/daily GH	1.01 (0.84; 1.22)	0.9022

Previously treated patients												
	Somapacitan			Daily GH			Comparison				ETR (95% CI)	P-value
	Baseline FSI, pmol/L	Last visit FSI, pmol/L	Relative Δ baseline	Baseline FSI, pmol/L	Last visit FSI, pmol/L	Relative Δ baseline						
REAL 2 (26 weeks)	90.2 (79.18)	90.5 (126.32)	0.89	77.1 (54.75)	83.0 (140.19)	0.87	Somapacitan vs daily GH				1.02 (0.78; 1.34)	0.8915
REAL Japan (52 weeks)	78.7 (64.6)	69.5 (57.5)	0.86	90.2 (64.8)	71.5 (45.3)	0.89	Somapacitan vs daily GH				0.97 (0.69; 1.35)	0.8393

	Daily GH/somapacitan^**a**^			Daily GH/daily GH^a^			Comparison				ETR (95% CI)	P-value
REAL 1 extension (week 34 to week 86)	101.6 (76.23)	82.1 (61.08)	0.80	111.2 (102.93)	69.6 (45.08)	0.69	Daily GH/somapacitan vs daily GH/daily GH				1.15 (0.94; 1.42)	0.1753

^**a**^These two groups are subgroups of the daily GH group in REAL 1. Patients receiving daily GH in the main phase were re-randomized at week 34 to receive either daily GH or once-weekly somapacitan in the extension phase. Baseline for these patients is week 34 (end of the main phase/start of the extension phase)

Baseline and last visit values are observed values shown as mean (SD). Relative changes are shown as ratios. Relative changes and ETRs were obtained using a mixed model of repeated measurements

Δ baseline, change from baseline; *CI* confidence interval, *ETR* estimated treatment ratio, *FSI* fasting serum insulin, *GH* growth hormone, *SD* standard deviation

The results of the subgroup analyses revealed that changes in glucose parameters (FPG, HbA1c, HOMA-IR and fasting serum insulin) in the three treatment arms were not influenced by sex, age, baseline fasting insulin, baseline glucose tolerance status or BMI (Supplementary Table S3).

### Previously treated patients (REAL 2, REAL Japan and REAL 1 extension)

In previously treated patients, the differences between the somapacitan and daily GH in changes from baseline in FPG were small and not statistically significant in any of the three trials (Fig. 1b, c, d). Similarly, the differences in HbA1c changes from baseline were small and not statistically significant between treatments in each trial (Fig. 2b, c, d).

Differences in changes from baseline in fasting insulin, HOMA-IR and HOMA-β were not statistically significant between somapacitan and daily GH in any of the trials (Fig. 3b, c, d, Table 3, Supplementary Table S2). Treatment periods were 26 weeks in REAL 2 and 52 weeks in REAL Japan and REAL 1 extension phase.

## Discussion

In these post hoc-defined analyses, we evaluated the long-term effects of somapacitan on glucose metabolism and insulin resistance in patients with AGHD in three phase 3 trials. In comparison with placebo and daily GH, somapacitan showed no clinically relevant adverse effects on glucose metabolism in any of the three complementary trials. In treatment-naïve patients, the effects of somapacitan and daily GH on these outcome measures were comparable after 86 weeks of treatment. Similarly, in patients previously treated with GH, there were no differences between treatments in these measures after 26 or 52 weeks of treatment. Furthermore, no new cases of diabetes were reported among patients treated with somapacitan. These results, from the largest data set available thus far to address the effects of a LAGH over 26 to 86 weeks of treatment, provide reassurance that glucose metabolism does not worsen when patients switch from daily GH to once-weekly somapacitan.

The effects of somapacitan and daily GH on glucose metabolism and insulin resistance were similar in all the trials, except that FPG, fasting serum insulin and HOMA-IR changes from baseline were higher for daily GH than somapacitan in treatment-naïve patients at week 34. These differences were not observed at week 86. The reason for a greater increase in FPG with daily GH compared with somapacitan at week 34 is unclear and may have been a chance finding or it might be possible that the acute and chronic effects of the treatments on glucose metabolism are slightly different. The kinetics of serum GH and IGF-I levels are different to those of daily GH; therefore, it is possible that detailed analyses may reveal variations between daily GH and somapacitan regarding their effects on glucose metabolism, although these differences may not be clinically relevant.

Several studies have suggested that during the early stages of GHRT, there is an initial deterioration in insulin sensitivity that can return to baseline values following longer term treatment, although results have been inconsistent. For example, when Cenci et al. assessed 14 patients with AGHD every 3 months for 5 years, they found that fasting glucose, insulin levels and insulin resistance did not change and that, despite an initial increase in frequency of abnormal glucose tolerance, mean 2-h oral glucose tolerance test glucose levels decreased between years 4 and 5 [26]. In a follow-up of 572 patients from the Hypopituitary Control and Complications Study treated with GH for 2.3–5.3 years, Woodmansee et al. reported that initiation of GH replacement in patients with AGHD was associated with a mild increase in FPG that often normalized spontaneously [9]. Sesmilo et al. studied 40 men with AGHD and observed increases in glucose levels, insulin levels and insulin-to-glucose ratios at 1 month [27]. Glucose and insulin levels subsequently decreased; however, at 18 months, the increase in glucose level (but not insulin level or insulin-to-glucose ratios) was maintained whereas HbA1c levels remained unchanged [27]. In a 5-year study of 118 adults with AGHD, Götherström et al. reported that blood glucose concentrations were increased throughout the study, whereas serum insulin concentration was not affected, and HbA1c level was lower at 5 years compared with baseline [28]. Results from a 33-month extension of a 9-month randomized clinical trial in 39 patients with AGHD demonstrated no change in HbA1c or plasma glucose after 42 months, although an increase in fasting serum insulin levels was reported [29]. As Berryman et al. have previously discussed, it is possible that these observed differences between studies on GHRT could be attributed to variations in patient selection [30]. For instance, discrepancies between studies regarding the inclusion or exclusion of patients with diabetes or varying degrees of insulin resistance may impact observations of the overall effect of GHRT on long-term insulin sensitivity.

The REAL1 trial presented an opportunity to investigate whether somapacitan poses a similar risk to patients during the early phase of GHRT. During the 34-week placebo-controlled phase, none of the glucose metabolism or insulin resistance measures changed during somapacitan treatment. In contrast, as described above, these parameters worsened in the daily GH treatment arm when compared to somapacitan. This effect appears to be transient as it was no longer present at the end of the extension phase, during which the effects of treatment following a switch from daily GH to somapacitan were compared. While the REAL1 trial results could be considered in line with previous reports that daily GH treatment may induce a transient deterioration in glucose metabolism, the observations from this placebo-controlled trial do not show the same for somapacitan.

It has been suggested that possible adverse effects of GH, which could lead to insulin resistance and hyperglycemia in the short term, might be counterbalanced by favorable concurrent changes in body composition [18]. Visceral fat is an important indicator of metabolic risk, and of cardiovascular disease and type 2 diabetes in particular [31]. A reduction in visceral fat and an increase in total lean body mass were indeed observed with longer-term use of both somapacitan and daily GH in the REAL 1 extension [20].

There have also been a few reports on the effects of LAGH preparations other than somapacitan on glucose metabolism in AGHD. Biller et al. reported no significant changes from baseline to 26 weeks in glucose parameters with the LAGH LB03002 [32]. In a follow-up to that study, 93 patients continued to receive open-label LB03002 for a total treatment period of 12 months [33]. There were no statistically significant changes from baseline to 12 months in mean FPG, HbA1c or fasting insulin in this group. Diabetes mellitus was reported in three patients (3.2%) [33]. Separately, Hoffman et al. reported no significant changes in glucose parameters after 32 weeks of treatment with Nutropin Depot (which has since been discontinued) or daily GH [34].

Strengths of this study include the fact that it provides an overview of data on the impact on glucose metabolism of a LAGH relative to that of daily GH. It is, to our knowledge, the first such study based on several complementary clinical trials of a LAGH and with treatment periods of up to 86 weeks. Each of the three studies described the use of somapacitan in a randomized, controlled, prospective trial, with similar endpoints being assessed throughout. Somapacitan was compared with daily GH in all three trials and also with placebo in REAL 1. A large number of patients from 20 countries over four continents were involved, reflecting a diverse geographical patient background [20–22].

Limitations include the fact that the analyses were based on separate studies in different locations and with slightly different study designs. Also, very few subjects (< 6%) had diabetes at baseline and all had mild disease, therefore the effects of somapacitan on glycemia in those with more severe diabetes (e.g., patients with HbA1c > 7%) cannot be inferred from these data.

## Conclusions

In this analysis of three studies comparing somapacitan with daily GH in the treatment of AGHD, the two GH preparations had similar effects on glucose parameters over the duration of the studies. These results provide reassurance that initiating GH replacement therapy with weekly administration of somapacitan does not incur any adverse effects on glucose metabolism relative to daily administration of GH. Furthermore, glucose metabolism did not worsen when patients were switched from daily GH to once-weekly somapacitan. Thus, somapacitan may provide a useful alternative to daily GH for patients with AGHD, with the need for less frequent injections expected to reduce treatment burden and cause less interference with daily life.

## Supplementary Information

Below is the link to the electronic supplementary material.

Supplementary file1 (DOCX 272 kb)

## Data Availability

The datasets analyzed during the current study are available from the corresponding author on reasonable request.
