# Supplementary material for: Weekly somapacitan had no adverse effects on glucose metabolism in adults with growth hormone deficiency
Source: Pituitary. 2022 Nov 15;26(1):57–72. doi: 10.1007/s11102-022-01283-3 (PMC9908671; doi:10.1007/s11102-022-01283-3)
Supplement: Supplementary file 1 — Supplementary file1 (DOCX 272 kb) [file 11102_2022_1283_MOESM1_ESM.docx]

**Supplementary material for:**

**Weekly somapacitan had no adverse effects on glucose metabolism in adults with growth hormone deficiency**

Yutaka Takahashi,^1^ Beverly MK Biller,^2^ Hidenori Fukuoka,^3^ Ken KY Ho,^4^ Michael Højby Rasmussen,^5^ Navid Nedjatian,^6^ Claus Sværke,^5^ Kevin CJ Yuen,^7^ Gudmundur Johannsson^8^

^1^ Department of Diabetes and Endocrinology, Nara Medical University, Kashihara, Japan

^2^ Neuroendocrine & Pituitary Tumor Clinical Center, Massachusetts General Hospital and Harvard Medical School, Boston, MA, USA

^3^ Division of Diabetes and Endocrinology, Kobe University Hospital, Kobe, Japan

^4^ Garvan Institute of Medical Research, St. Vincent’s Hospital, and UNSW Sydney, Sydney, Australia

^5^ Global Development, Novo Nordisk A/S, Søborg, Denmark

^6^ Global Medical Affairs – Rare Endocrine Disorders, Novo Nordisk Health Care AG, Zürich, Switzerland

^7^ Barrow Pituitary Center, Barrow Neurological Institute and St. Joseph's Hospital and Medical Center, University of Arizona College of Medicine and Creighton School of Medicine, Phoenix, AZ, USA

^8^ Institute of Medicine, Sahlgrenska Academy, University of Göteborg, and Department of Endocrinology, Sahlgrenska University Hospital, Göteborg, Sweden

**Corresponding author:** Yutaka Takahashi
Address: 840 Shijo-cho, Kashihara, Nara 634-8522, Japan
Tel number: 81-744-22-3051
Email: takahash@naramed-u.ac.jp

**Supplementary Figure 1** Trial designs for **a**) REAL 1, **b**) REAL 2 and **c**) REAL Japan [1–3].

**a) REAL 1 and extension**


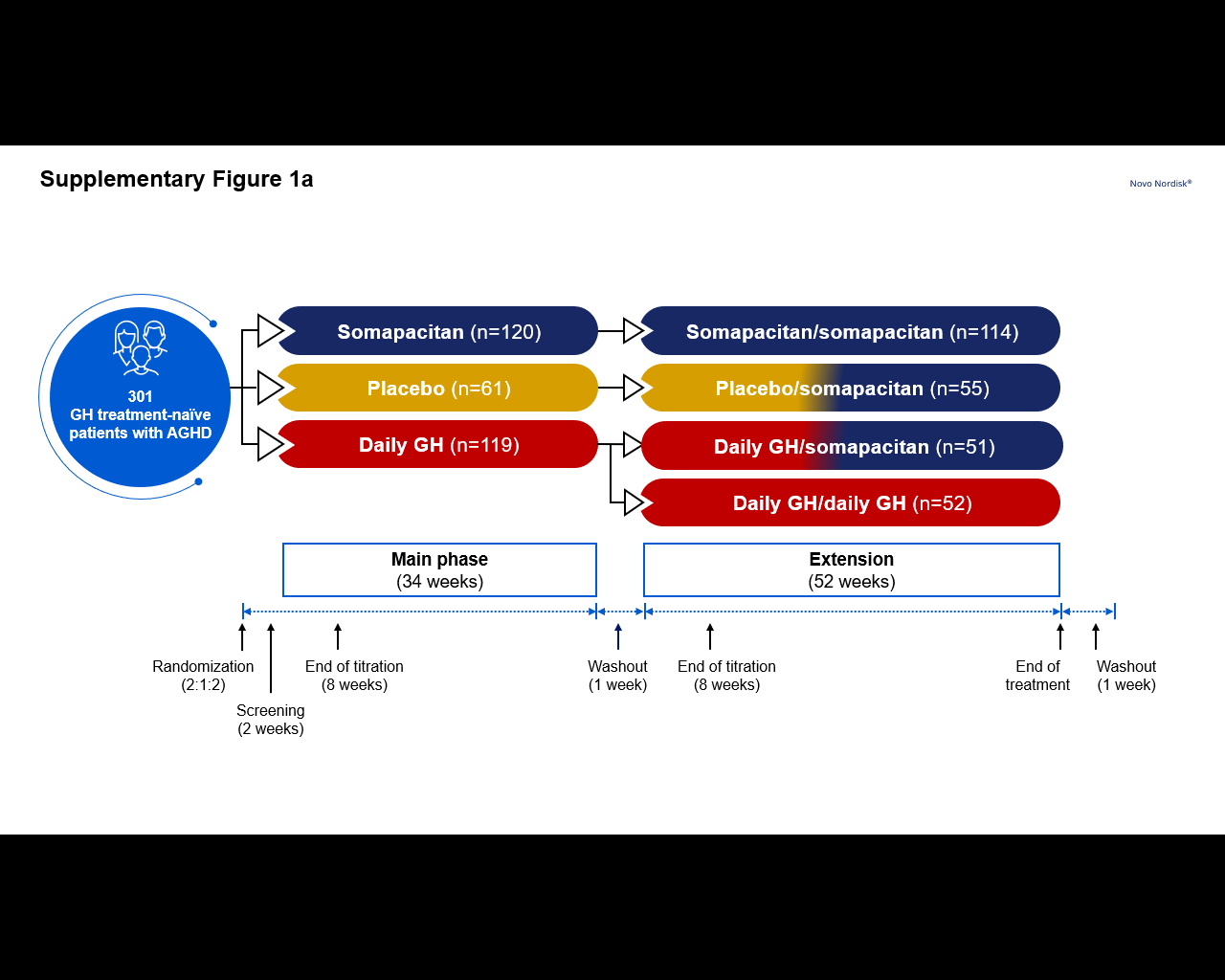


**Blood samples were taken as follows: on the day of somapacitan dosing (week 8 and 43); 1 day after dosing (week 25 and 64); or 4 days after dosing (week 17, 34, 54, 76 and 87). Adapted from Fig. 1 of Johannsson G et al. Once-weekly somapacitan is effective and well tolerated in adults with GH deficiency: a randomized phase 3 trial. J Clin Endocrinol Metab 2020;105:e1358-1376. © 2020 Oxford University Press.
AGHD, adult growth hormone deficiency, GH, growth hormone.**

**b) REAL 2**


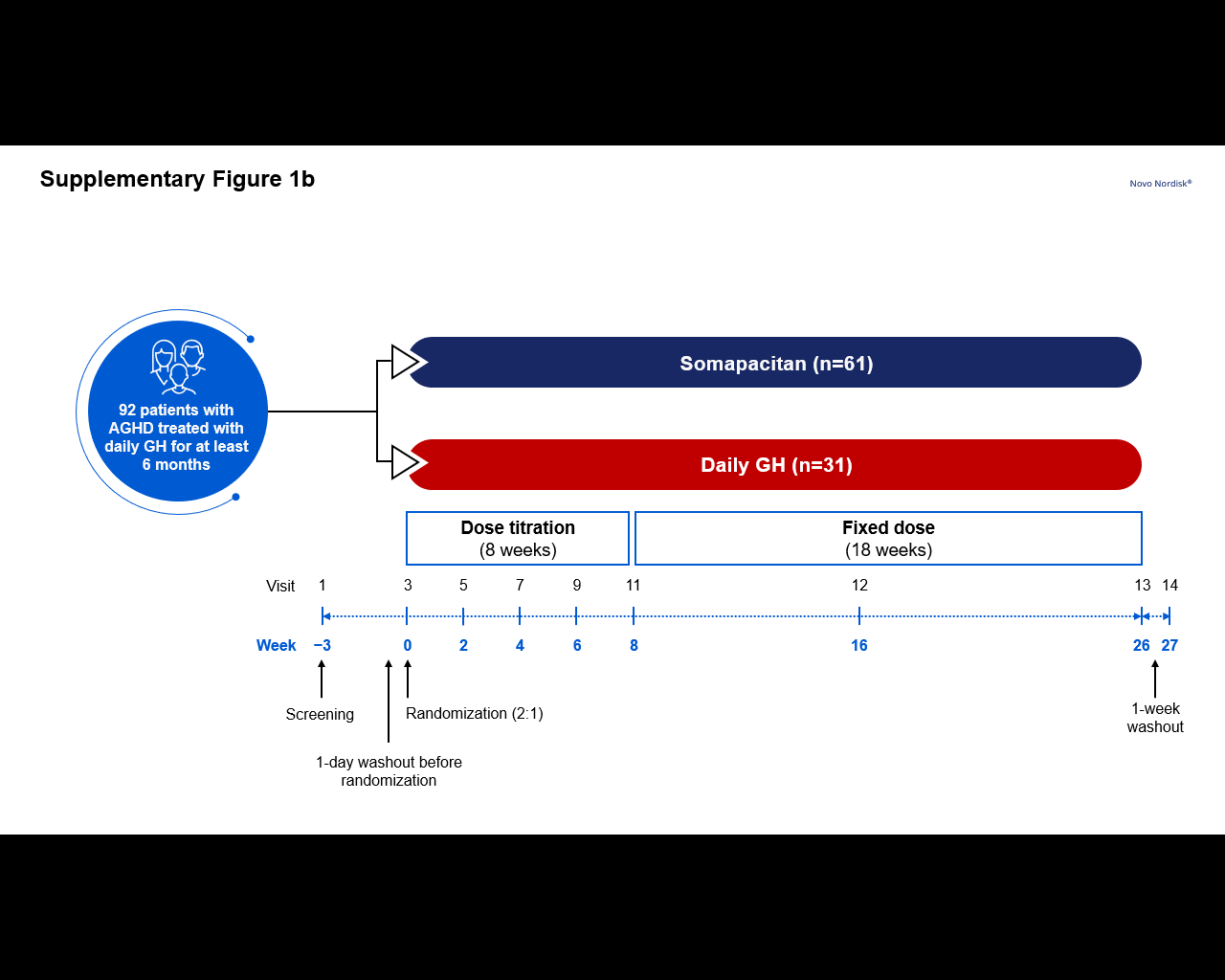


**Blood samples were taken on the day of somapacitan dosing (week 8); or 4 days after dosing (week 17 and 26). Adapted from supplementary Fig. 1 of Johannsson G et al. Safety and convenience of once-weekly somapacitan in adult GH deficiency: a 26-week randomized, controlled trial. Eur J Endocrinol 2018;178:491-499. © 2018 The authors. Published by Bioscientifica Ltd.
AGHD, adult growth hormone deficiency, GH, growth hormone.**

**c) REAL Japan**


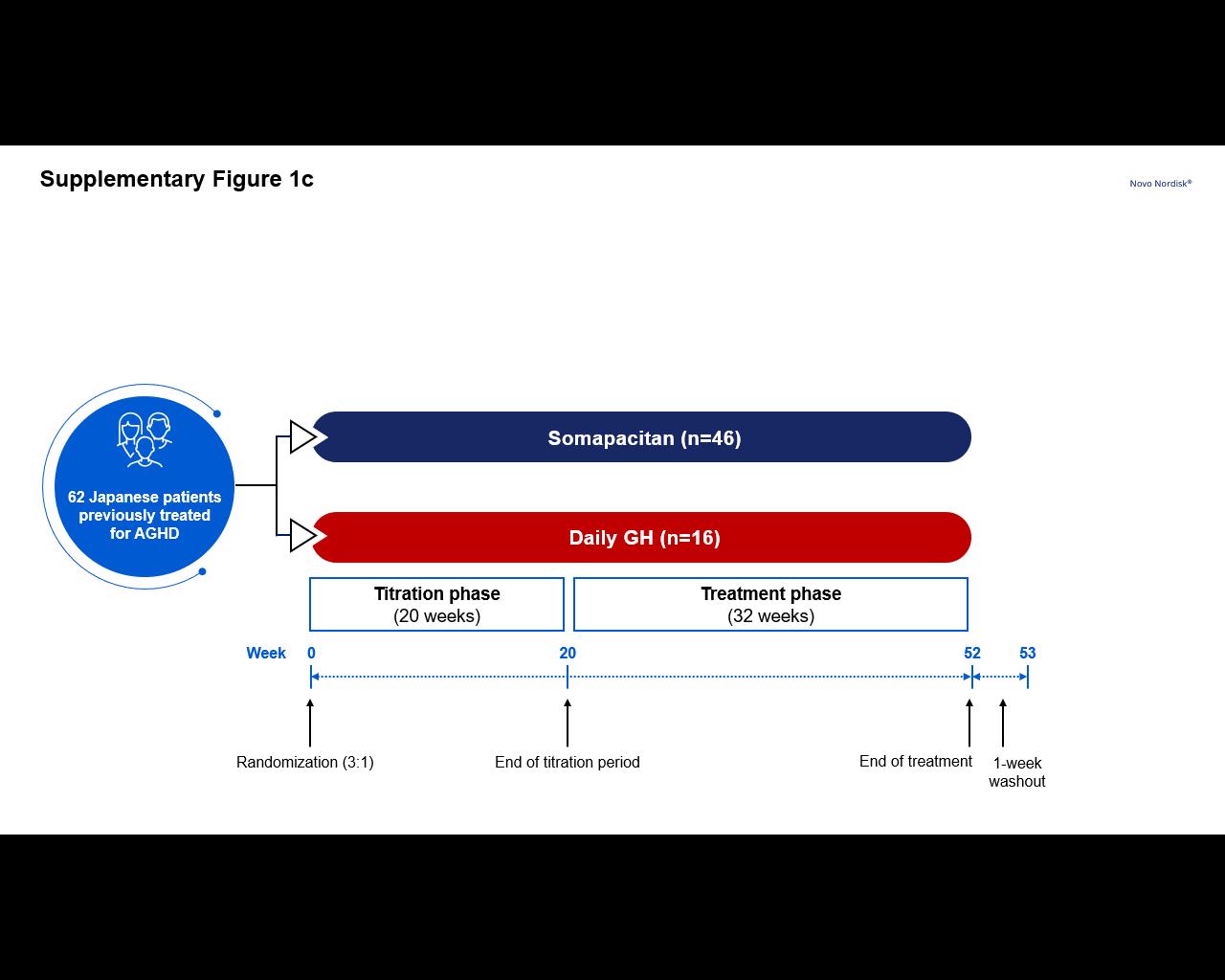


**Blood samples were taken 3 days after somapacitan dosing (week 3, 7 and 19) or 4 days after dosing (week 33 and 52).** Adapted from Fig. 1 of Otsuka F et al. Similar safety and efficacy in previously treated adults with growth hormone deficiency randomized to once-weekly somapacitan or daily growth hormone. Clin Endocrinol 2020;93:620-628. © 2020 The Authors. Published by John Wiley & Sons Ltd.

AGHD, adult growth hormone deficiency; GH growth hormone.

**Supplementary Table 1** Baseline and last visit IGF-I SDS

| **Treatment-naïve patients** | | | | | | |
| --- | --- | --- | --- | --- | --- | --- |
|  | **Somapacitan** | | **Daily GH** | | **Placebo** | |
|  | Baseline  IGF-I SDS | Last visit  IGF-I SDS | Baseline  IGF-I SDS | Last visit  IGF-I SDS | Baseline  IGF-I SDS | Last visit  IGF-I SDS |
| **REAL 1 main phase  (34 weeks)** | −2.58  (1.21) | −0.17  (1.25) | −2.53  (1.18) | −0.23  (1.11) | −2.68  (1.29) | −2.62  (1.33) |
|  |  |  |  |  |  |  |
| **REAL 1 main phase  + extension  (86 weeks)** | −2.59  (1.20) | −0.24  (1.29) | −2.43  (1.25) | −0.26  (1.34) | – | – |
| **Previously treated patients** | | | | | | |
|  | **Somapacitan** | | **Daily GH** | |  |  |
|  | Baseline  IGF-I SDS | Last visit  IGF-I SDS | Baseline  IGF-I SDS | Last visit  IGF-I SDS |  |  |
| **REAL 2  (25 weeks)** | 0.28  (1.50) | 0.22  (0.89) | 0.91  (1.24) | 0.35  (0.82) |  |  |
| **REAL Japan  (52 weeks)** | 0.64  (0.72) | 0.61  (0.68) | 0.88  (0.82) | 0.52  (0.57) |  |  |
|  | **Daily GH/somapacitan^a^** | | **Daily GH/daily GH^a^** | |  |  |
| **REAL 1 extension  (week 34 to week 86)** | −0.41  (1.05) | −0.44  (1.17) | 0.11  (0.86) | −0.26  (1.34) |  |  |

^a^These two groups are subgroups of the daily GH group in REAL 1. Patients receiving daily GH in the main phase were re-randomized at week 34 to receive either daily GH or once-weekly somapacitan in the extension phase. Baseline for these patients is week 34 (end of the main phase/start of the extension phase).

Baseline and last visit values are observed values shown as mean (SD).

GH, growth hormone; IGF-I, insulin-like growth factor I; SD, standard deviation; SDS, standard deviation score.

**Supplementary Table 2** Changes from baseline in HOMA-β, a measure of steady-state cell function, and comparisons (estimated treatment ratio) between treatment groups

| **Treatment-naïve patients** | | | | | | | | | | | | |
| --- | --- | --- | --- | --- | --- | --- | --- | --- | --- | --- | --- | --- |
|  | **Somapacitan** | | | **Daily GH** | | | **Placebo** | | | **Comparison** | **ETR  (95% CI)** | ***P*-value** |
|  | Baseline HOMA-β, % | Last visit HOMA-β,  % | Relative Δ baseline | Baseline HOMA-β, % | Last visit HOMA-β, % | Relative Δ baseline | Baseline HOMA-β,  % | Last visit HOMA-β,  % | Relative Δ baseline |  |  |  |
| **REAL 1 main phase  (34 weeks)** | 198.53 (198.63) | 246.23 (198.63) | 1.26 | 188.61 (255.48) | 226.80 (181.77) | 1.33 | 165.97 (147.49) | 192.51 (186.29) | 1.11 | Somapacitan  vs placebo | 1.13  (0.94; 1.35) | 0.1858 |
|  |  |  |  |  |  |  |  |  |  | Somapacitan  vs daily GH | 0.95  (0.82; 1.10) | 0.4704 |
| **REAL 1 main phase  + extension (86 weeks)** | 198.53 (198.63) | 185.26 (172.0) | 0.98 | 165.59 (107.53) | 159.99 (119.36) | 0.96 | - | - | - | Somapacitan/ somapacitan vs daily GH/daily GH | 1.02  (0.85; 1.22) | 0.8582 |
| **Previously treated patients** | | | | | | | | | | | | |
|  | **Somapacitan** | | | **Daily GH** | | | **Comparison** | | | | **ETR  (95% CI)** | ***P*-value** |
|  | Baseline HOMA-β,  % | Last visit HOMA-β,  % | Δ baseline | Baseline HOMA-β,  % | Last visit HOMA-β, % | Δ baseline |  |  |  |  |  |  |
| **REAL 2  (25 weeks)** | 162.18 (116.93) | 190.71 (201.37) | 1.05 | 139.13 (89.83) | 147.85 (133.24) | 1.06 | Somapacitan vs daily GH | | | | 0.99  (0.77; 1.29) | 0.9674 |
| **REAL Japan  (52 weeks)** | 131.9 (102.91) | 113.62 (84.27) | 0.84 | 142.79 (82.05) | 112.69 (57.61) | 0.85 | Somapacitan vs daily GH | | | | 0.99  (0.73; 1.34) | 0.9492 |
|  | **Daily GH/somapacitan^a^** | | | **Daily GH/daily GH^a^** | | | **Comparison** | | | | **ETR (95% CI)** | ***P*-value** |
| **REAL 1 extension  (week 34 to week 86)** | 228.23 (153.45) | 199.95 (163.63) | 0.84 | 241.47 (216.57) | 159.99 (119.36) | 0.69 | Daily GH/somapacitan vs daily GH/daily GH | | | | 1.21  (0.99; 1.48) | 0.0661 |

^a^These two groups are subgroups of the daily GH group in REAL 1. Patients receiving daily GH in the main phase were re-randomized at week 34 to receive either daily GH or somapacitan in the extension phase. Baseline for these patients is week 34 (end of the main phase/start of the extension phase).

Baseline and last visit values are observed values shown as mean (SD). Relative changes are shown as ratios. Relative changes and ETRs were obtained using a mixed effects model.

Δ baseline, change from baseline; CI, confidence interval; HOMA-β, steady state beta-cell function; ETR, estimated treatment ration; GH, growth hormone; SD, standard deviation.

**Supplementary Table 3** Estimated treatment differences/ratios between somapacitan and daily GH for glucose parameters at week 34 according to risk factors: treatment-naïve patients in REAL 1 main phase

|  | | **Comparison of estimated treatment difference/ratio: somapacitan vs daily GH** | | | |
| --- | --- | --- | --- | --- | --- |
|  |  | **FPG  ETD [95% CI]** | **HbA1c ETD [95% CI]** | **HOMA-IR**  **ETR [95% CI]** | **Fasting serum insulin ETR [95% CI]** |
| **Overall** |  | –0.16 [–0.30; –0.03] | 0.00 [–0.08; 0.08] | 0.80 [0.69; 0.94] | 0.85 [0.73; 0.99] |
| **Sex** | Male | –0.17 [–0.33; –0.01] | 0.07 [–0.06; –0.19] | 0.78 [0.62; 0.98] | 0.81 [0.64;1.02] |
|  | Female | -0.17 [–0.38; 0.05] | –0.06 [–0.16; 0.04] | 0.83 [0.67; 1.02] | 0.88 [0.72; 1.08] |
| **Age** | <40 years | –0.12 [–0.32; 0.07] | 0.04 [–0.08; 0.15] | 0.74 [0.57; 0.97] | 0.76 [0.58; 0.99] |
|  | 40–59 years | –0.18 [–0.40; 0.03] | –0.04 [–0.16; 0.08] | 0.80 [0.61; 1.04] | 0.85 [0.67; 1.08] |
|  | ≥60 years | –0.04 [–0.39; 0.31] | 0.04 [–0.20; 0.28] | 1.01 [0.79; 1.29] | 1.07 [0.85; 1.35] |
| **Fasting serum insulin** | Hypoinsulinemia | –0.16 [–0.61; 0.29] | 0.13 [–0.24; 0.50] | 0.35 [0.11; 1.05] | 0.35 [0.08; 1.43] |
|  | Normal  (≥14–≤208 pmol/L) | –0.20 [–0.34; –0.06] | –0.01 [–0.10; 0.07] | 0.80 [0.67; 0.94] | 0.83 [0.72; 0.97] |
|  | Hyperinsulinemia | 0.46 [–0.90; 1.83] | 0.07 [–0.80; 0.93] | 2.25 [0.93; 5.47] | 2.39 [1.06; 5.39] |
| **Baseline BMI** | <30 kg/m^2^ | –0.15 [–0.32; 0.02] | 0.01 [–0.08; 0.10] | 0.80 [0.66; 0.97] | 0.84 [0.69; 1.02] |
|  | ≥30 kg/m^2^ | –0.19 [–0.42; 0.04] | –0.07 [–0.23; 0.09] | 0.76 [0.58; 0.99] | 0.80 [0.62; 1.03] |
| **Glucose tolerance** | Normal | –0.11 [–0.25; 0.03] | –0.01 [–0.10; 0.07] | 0.77 [0.63; 0.93] | 0.80 [0.66; 0.96] |
|  | Prediabetes | –0.26 [–0.56; 0.03] | 0.03 [–0.15; 0.21] | 0.89 [0.67; 1.19] | 0.94 [0.72; 1.22] |

ETRs and ETDs were obtained using a mixed effects model.

BMI, body mass index; CI, confidence interval; ETD, estimated treatment difference; ETR, estimated treatment ratio; FPG, fasting plasma glucose; GH, growth hormone; HbA1c, glycated hemoglobin; HOMA-IR, homeostasis model assessment insulin resistance**.**

**References**

1. Johannsson, G., Gordon, M.B., Rasmussen, M.H., Håkonsson, I.H., Karges, W., Sværke, C., Tahara, S., Takano, K., Biller, B.M.K.: Once-weekly somapacitan is effective and well tolerated in adults with GH deficiency: a randomized phase 3 trial. J Clin Endocrinol Metab. 105(4):e1358-1376 (2020). <https://doi.org/10.1210/clinem/dgaa049>
2. Johannsson, G., Feldt-Rasmussen, U., Håkonsson, I.H., Biering, H., Rodien, P., Tahara, S., Toogood, A., Rasmussen, M.H., REAL 2 Study Group: Safety and convenience of once-weekly somapacitan in adult GH deficiency: a 26-week randomized, controlled trial. Eur J Endocrinol. 178(5):491-499 (2018). <https://doi.org/10.1530%2FEJE-17-1073>
3. Otsuka, F., Takahashi, Y., Tahara, S., Ogawa, Y., Højby Rasmussen, M., Takano, K.: Similar safety and efficacy in previously treated adults with growth hormone deficiency randomized to once-weekly somapacitan or daily growth hormone. Clin Endocrinol. 93(5):620-628 (2020). <https://doi.org/10.1111/cen.14273>
